# Segmenting beyond the imaging data: creation of anatomically valid edentulous mandibular geometries for surgical planning using artificial intelligence

**DOI:** 10.1007/s00784-025-06471-6

**Published:** 2025-10-11

**Authors:** Stefan Raith, Tobias Pankert, Srikrishna Jaganathan, Kristina Pankert, Hyun Lee, Florian Peters, Frank Hölzle, Ali Modabber

**Affiliations:** 1 Department of Oral and Maxillofacial Surgery, RWTH Aachen University Hospital, Pauwelsstraße 30, 52074 Aachen, Germany; 2 Inzipio GmbH, Krantzstr. 7 Building 80, 52070 Aachen, Germany

**Keywords:** Mandible, Segmentation, Deep learning, Convolutional neural networks, Computed tomography, Virtual surgical planning, Medical image analysis, Reconstruction

## Abstract

**Background and objectives:**

Mandibular reconstruction following continuity resection due to tumor ablation or osteonecrosis remains a significant challenge in maxillofacial surgery. Virtual surgical planning (VSP) relies on accurate segmentation of the mandible, yet existing AI models typically include teeth, making them unsuitable for planning of autologous transplants dimensions aiming for reconstructing edentulous mandibles optimized for dental implant insertion. This study investigates the feasibility of using deep learning-based segmentation to generate anatomically valid, toothless mandibles from dentate CT scans, ensuring geometric accuracy for reconstructive planning.

**Methods:**

A two-stage convolutional neural network (CNN) approach was employed to segment mandibles from computed tomography (CT) data. The dataset (*n* = 246) included dentate, partially dentate, and edentulous mandibles. Ground truth segmentations were manually modified to create Class III (moderate alveolar atrophy) and Class V (severe atrophy) models, representing different degrees of post-extraction bone resorption. The AI models were trained on the original (O), Class III (Cl. III), and Class V (Cl. V) datasets, and performance was evaluated using Dice similarity coefficients (DSC), average surface distance, and automatically detected anatomical curvatures.

**Results:**

AI-generated segmentations demonstrated high anatomical accuracy across all models, with mean DSCs exceeding 0.94. Accuracy was highest in edentulous mandibles (DSC 0.96 ± 0.014) and slightly lower in fully dentate cases, particularly for Class V modifications (DSC 0.936 ± 0.030). The caudolateral curve remained consistent, confirming that baseline mandibular geometry was preserved despite alveolar ridge modifications.

**Conclusions:**

This study confirms that AI-driven segmentation can generate anatomically valid edentulous mandibles from dentate CT scans with high accuracy. The innovation of the work is the precise adaptation of alveolar ridge geometry, making it a valuable tool for patient-specific virtual surgical planning in mandibular reconstruction.

## Introduction

The reconstruction of the mandible after continuity resection due to tumor ablation or medication-related necrosis is one of the most challenging tasks in maxillofacial surgery [1]. While there are different alternatives, the current gold standard is autologous transplantation of bone flaps from the fibula, the iliac crest or the scapula [2]. Since the fibula is a relatively thin bone, it provides only partial height for complete restoration of the alveolar crest—often necessitating techniques such as the so-called double-barrel method [3]. Unlike the tube-shaped fibula, the transplants from iliac crest and scapula offer sufficient bone material for a whole reconstruction of the alveolar process of the mandible making them an advantageous choice for functional rehabilitation with implant based dental prostheses.

Virtual surgical planning of mandibular reconstruction has shown significant benefits in comparison to free hand surgery [4] and can nowadays be considered the inevitable state-of-the-art for transplant planning, at least in complex cases. For the accurate planning of the transplant geometry, the shape of the defect, i.e. the structure of the bone that needs to be reconstructed, needs to be known for optimal planning. Applications for the estimation of the shape of a missing part of the mandible have been introduced leveraging the potential of statistical shape models [5, 6], however these models either leave the dentition out of scope [6] or preselect only dentate mandibles [5]. Yet from a surgical perspective, the task should be phrased as generation of a geometry that resembles an edentulous mandible that is optimally suited for subsequent insertion of dental implants and thus be used as a target geometry for reconstructive surgery.

In recent years, a multitude of different approaches using artificial intelligence, mostly implemented as deep learning using convolutional neural networks as their basis, were proposed for mandibular segmentation [7–9]. Typically, these approaches use collectives of mandibles with varying dental status [7, 9, 10]. While most of these models are trained on segmenting the mandible in conjunction with the lower teeth [9], others are specifically trained to distinguish the mandibular bone from other anatomical regions, such as the crowns of the teeth [11], potentially with the ability to detect every tooth separately [12–16], thus making these mandibles geometries with empty alveolar cavities, resembling the state directly after dental extraction [17]. Thus, neither of these models provides the geometry of an edentulous mandible, that would be the aim of reconstructive surgery.

Edentulous jaw segmentation for subsequent implantation planning has so far only been applied to regionally limited fields of view [18], with the focus of single implant planning. However, no approaches of estimating the geometry of an edentulous alveolar ridge based on imaging data from dentate mandibles have been investigated yet.

Hence, this is to the best of our knowledge the first work that deals with that component of the process chain in surgical reconstruction of the mandible with bony transplants. While the approach for automated segmentation is derived from an established 3D UNet, the innovative aspect of this work lies in the use of artificial intelligence in the virtual modelling of clinically relevant edentulous geometries from dentate scans to support preoperative planning in reconstructive surgery.

To achieve that, original segmentations of the lower jaw were modified to reflect different degrees of edentulous status and even atrophy as classified by Cawood and Howell [19]. These modified segmentations were then used to train segmentation models capable of segmenting different atrophic versions of the mandible bone from CT data.

In a more generalized way, the exploratory character of this work aims to investigate whether it is possible to derive anatomically valid shapes that still carry features of an individual anatomy but have alterations to specific features of these structures while maintaining the individual shape characteristics.

The current work investigates the following three hypotheses, with the first being the primary endpoint of the study:

- (H1) AI models are capable of estimating geometrically valid geometries of toothless mandibles, even based on imaging data of dentate mandibles.
- (H2) That these geometries have sufficient accuracy at the ascending branches and the basal parts to be used as a basis for patient individual surgical planning.
- (H3) Overall accuracy for the original data is higher than for the modified ones.

## Results

### Qualitative results

**Fig. 1:**
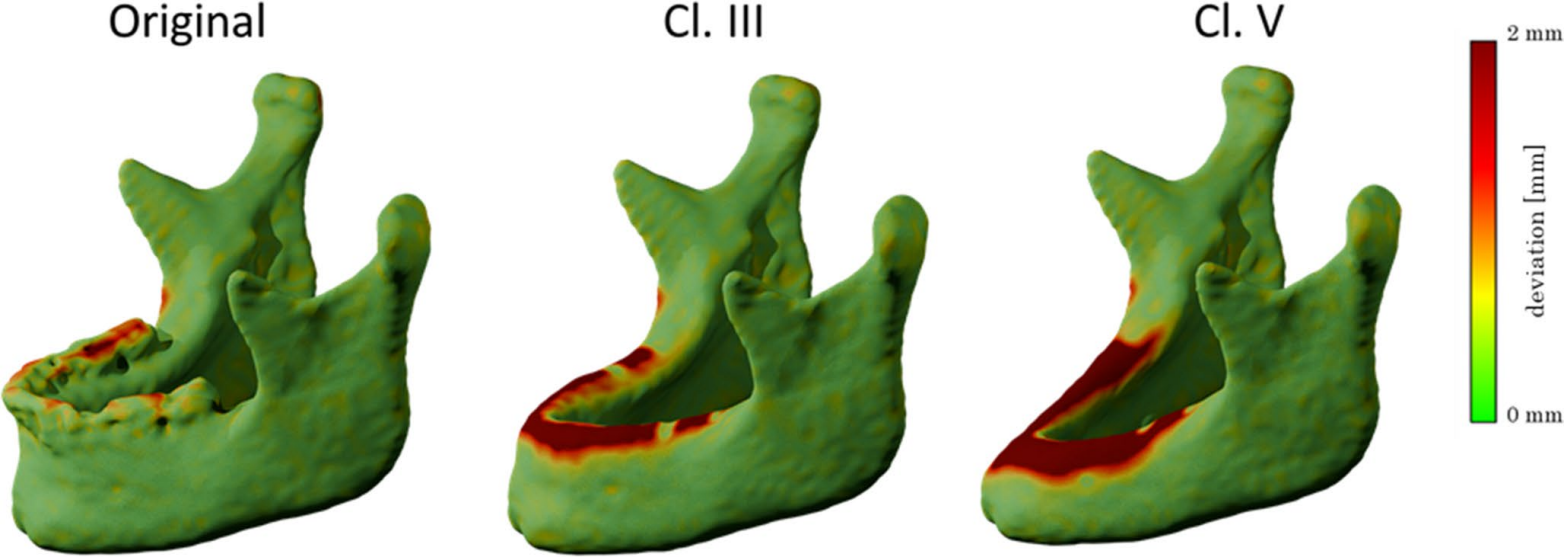
3D surface comparison of predictions of the different models against the original ground truths. Note the generally valid surfaces in large regions of the mandible, e.g. at the ascending branches and the body of the mandible. The teeth are less accurately segmented in the so-called ground truths; thus, the original model shows weaker performance in this region. The other two models, Cl. III and Cl. V, show a valid geometry of the alveolar crest and a comparably accurate geometry apart from that region

To visually assess the performance of the proposed AI-based segmentation approach, the predicted models for the three dataset variations—Original (O), Class III (Cl. III), and Class V (Cl. V) can be visually compared against their respective manually generated ground truths. Representative 3D surface renderings of the segmentations illustrate the ability of the AI models to reconstruct the overall mandibular structure while accurately adapting the alveolar crest according to the desired anatomical modifications (Fig. 1).

Across all test cases, the predicted segmentations as they were generated by the AI models demonstrated high anatomical validity, particularly in regions not subjected to modification, such as the mandibular body, ascending rami, condyles, and gonial angles. These structures were preserved across all variations, as expected. In contrast, the alveolar crest region exhibited controlled modifications in Cl. III and Cl. V segmentations, effectively mimicking edentulous mandibles with varying degrees of atrophy.

**Fig. 2:**
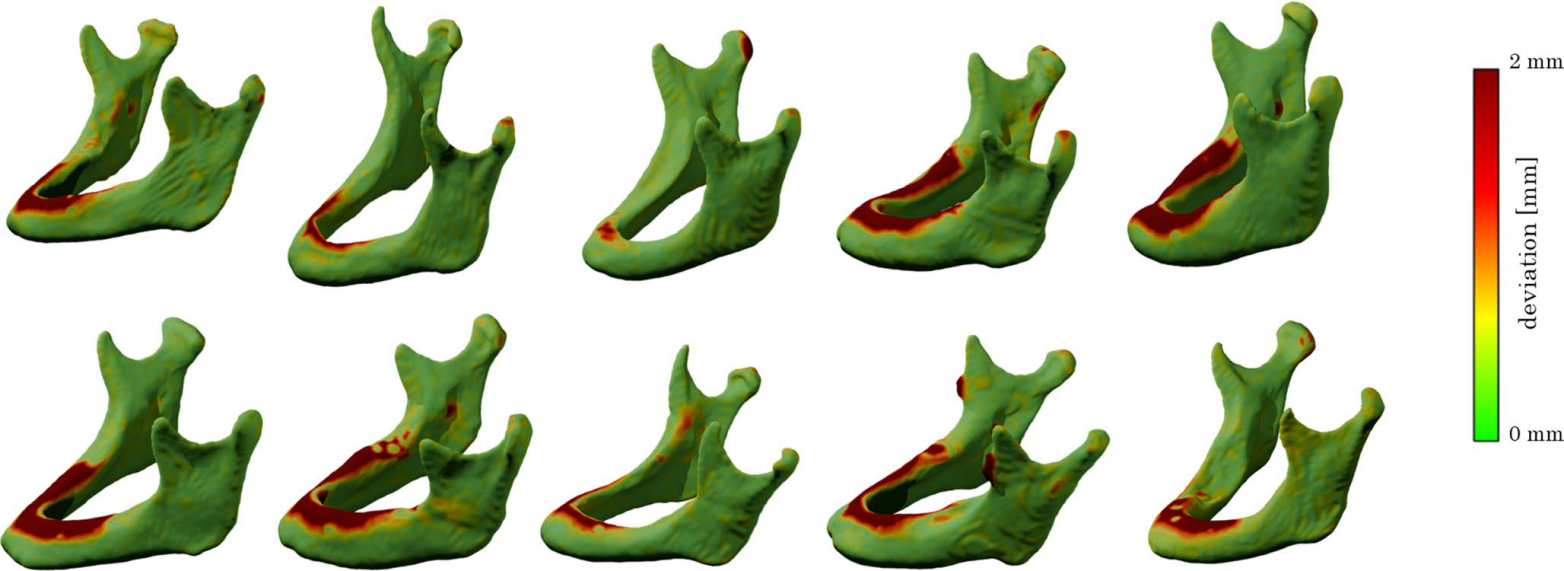
Comparison between predicted Cl. V and original ground truths. Geometries of the alveolar crests are always anatomically correct. Mandibular bodies and ascending branches are consistently captured with sufficient accuracy

**Fig. 3:**
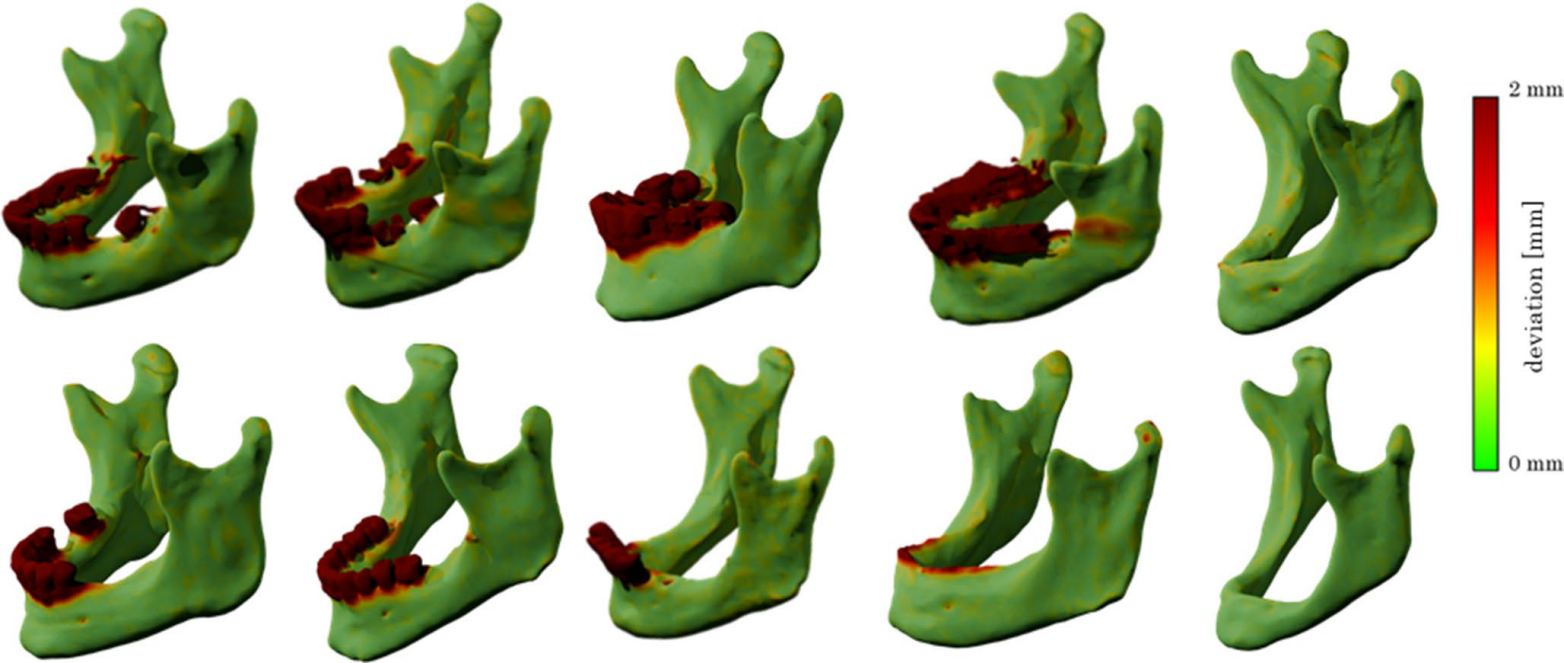
Comparison between original ground truths and predicted Cl. III. The comparison shows that for Cl. III predictions, only dental geometries were removed, leaving the continuous mandibular anatomy intact. For edentulous mandibles, their geometry was not altered significantly, as desired

Notably, Cl. III predictions accurately represented the removal of teeth while maintaining a continuous alveolar ridge (Fig. 2), whereas Cl. V predictions effectively demonstrated a resorption pattern, consistent with atrophic mandibular characteristics. Importantly, the model did not introduce unintended distortions in surrounding structures, confirming the segmentation’s reliability for reconstructive surgical planning (Fig. 3).

The qualitative results confirm that the AI-based segmentation approach successfully removes dentition in a controlled and anatomically valid manner, preserving essential mandibular structures while modifying the alveolar crest according to predefined anatomical classifications. These findings support the feasibility of using AI-generated edentulous mandibular models for patient-specific virtual surgical planning, potentially improving the accuracy and efficiency of bony transplant procedures.

### Quantitative results

All data groups were tested for normal distribution using the Shapiro–Wilk test and found not to be normally distributed (*p* < 0.05). Consequently, we employed the Wilcoxon signed-rank test for all pairwise comparisons.

#### Dice Coefficients

**Fig. 4:**
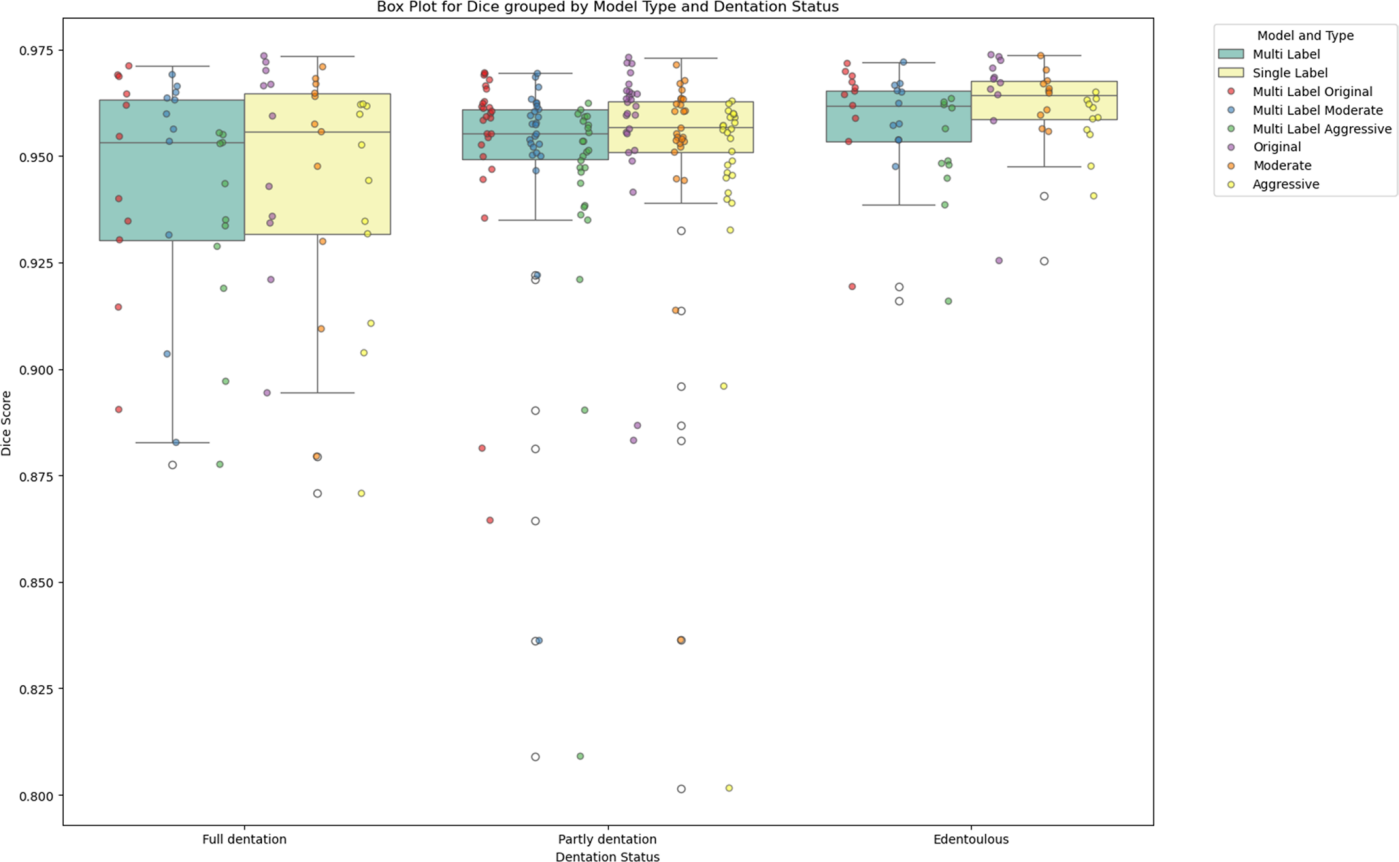
Visualization of Dice scores for the different groups of evaluations

The findings indicate that edentulous cases consistently achieve the highest performance across all models, with values ranging from 0.950 to 0.964, however this finding shows only to be significant for comparisons of the edentulous group toward partly dentate. Class V models show a slight decline in performance compared to the Original and Class III models, particularly in full and partly dentation cases. Multi-label models exhibit similar trends, maintaining strong results but with a minor drop, though statistically significant due to the nature of paired comparisons (*p* < 1e-8). An overview can be found in Table 1; Fig. 4.

**Table 1:** Dice coefficients of similarity distributed in the three categories and according to dental status

Model	Full Dentation (*n* = 11)	Partly Dentate (*n* = 26)	Edentulous (*n* = 11)	Overall
Single Label Original	0.949 ± 0.026	0.955 ± 0.022	0.964 ± 0.014	0.956 ± 0.022
Single Label Class III	0.947 ± 0.029	0.952 ± 0.026	0.964 ± 0.006	0.953 ± 0.024
Single Label Class V	0.936 ± 0.030	0.944 ± 0.032	0.957 ± 0.007	0.945 ± 0.028
Multi Label Original	0.945 ± 0.026	0.952 ± 0.025	0.961 ± 0.015	0.953 ± 0.023
Multi Label Cl. III	0.947 ± 0.029	0.952 ± 0.025	0.961 ± 0.007	0.953 ± 0.023
Multi Label Cl. V	0.932 ± 0.025	0.942 ± 0.031	0.950 ± 0.014	0.941 ± 0.027

#### Average surface distance

The results show that Average Surface Distance (ASD) values are generally higher for dentate cases (full and partly dentation) compared to edentulous cases across all models, indicating significantly better performance in edentulous scenarios (*p* < 0.05). Within dentate cases, Class V models, both Single Label and Multi-Label, tend to have higher ASD values than Original and Class III models, suggesting slightly lower performance. Specifically, in full dentation, Multi-Label Cl. V exhibits the highest ASD (0.567 ± 0.307), while Single Label Original shows the lowest (0.438 ± 0.324), both findings with statistical significance (*P* < 0.05). In edentulous cases, all models show improved ASD, with Class III Single Label achieving the lowest value (0.177 ± 0.054), and Multi-Label Cl. V having a relatively higher value (0.275 ± 0.133) among edentulous results. Multi-label models generally show slightly higher ASD values than their single-label counterparts across all dentation categories, though the differences are not substantial, they are still statistically significant due to the nature of paired comparisons (*p* < 1e-12). An overview can be found in Table 2.

**Table 2:** Average surface distance

Model	Full Dentation (*n* = 11)	Partly Dentate (*n* = 26)	Edentulous (*n* = 11)	Overall
Single Label Original	0.438 ± 0.324	0.327 ± 0.210	0.214 ± 0.260	0.327 ± 0.257
Single Label Class III	0.459 ± 0.354	0.352 ± 0.229	0.177 ± 0.054	0.337 ± 0.254
Single Label Class V	0.517 ± 0.325	0.394 ± 0.2504	0.224 ± 0.080	0.383 ± 0.259
Multi Label Original	0.482 ± 0.323	0.362 ± 0.219	0.252 ± 0.269	0.364 ± 0.263
Multi Label Cl. III	0.460 ± 0.340	0.363 ± 0.215	0.203 ± 0.064	0.349 ± 0.241
Multi Label Cl. V	0.567 ± 0.307	0.427 ± 0.2682	0.275 ± 0.133	0.425 ± 0.269

#### Curvature metrics

**Fig. 5:**
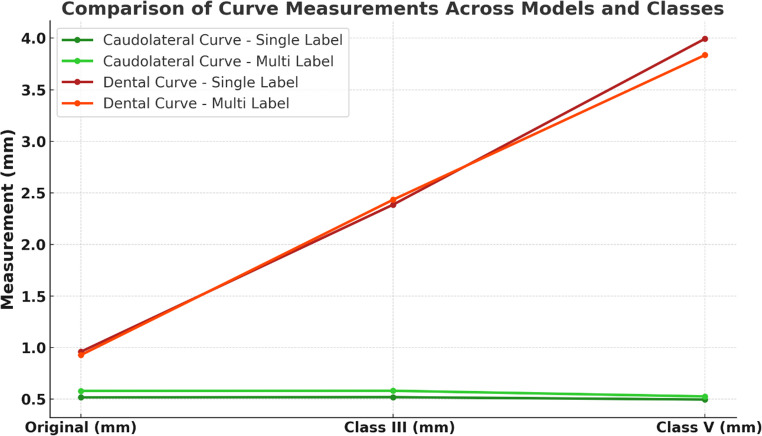
Illustrative chart of the two different curve metrics for the different groups

A detailed analysis of the caudolateral curve confirms that the basal contour of the mandible remained unchanged across all segmentation variations (Fig. 7). This observation aligns with anatomical expectations, as the inferior border of the mandible should not be affected by the removal of dental structures. The preservation of this contour supports the anatomical validity of the AI-generated modifications and suggests that these models can be reliably integrated into the workflow of virtual surgical planning for reconstructive procedures. The dental curve, however, is providing quantitative evidence that the dentate regions and the alveolar crest, respectively, do change with respect to the original datasets (Table 3; Fig. 5).

**Table 3:** Numeric values of the different curve metrics

Curve	Model	Original (mm)	Class III (mm)	Class V (mm)
Caudolateral Curve	Single Label	0.517	0.519	0.496
Caudolateral Curve	Multi Label	0.580	0.581	0.526
Dental Curve	Single Label	0.960	2.385	3.994
Dental Curve	Multi Label	0.928	2.433	3.836

## Discussion

This study demonstrates that artificial intelligence can generate anatomically valid, toothless mandible geometries, even based on medical imaging data of dentate mandibles.

The first main hypothesis could be confirmed, as the proposed approach may be integrated into a process chain of digital surgical planning of reconstructive surgery (H1). The ability to automatically remove teeth and adjust the alveolar ridge has significant implications for virtual surgical planning (VSP) in mandibular reconstruction. Traditionally, manual segmentation of the edentulous mandible is a time-consuming process requiring expert input. The proposed AI-driven approach automates this process, ensuring standardized, reproducible, and anatomically consistent modifications in a fraction of the time. This could streamline preoperative planning workflows, reducing the burden on clinicians and improving surgical precision. Although threshold-based segmentation and commercial AI tools can extract mandibular geometry, they cannot generate edentulous ridge contours with anatomically valid clinical classifications (e.g., Class III and V). Thus, the proposed method fills a clinical gap by enabling anatomically controlled virtual reduction of the alveolar ridge for the purpose of preoperative planning in reconstructive surgery.

The segmentation results showed high anatomical accuracy, with Dice similarity coefficients consistently exceeding 0.94 across all models. The highest performance was observed in edentulous mandibles (DSC 0.964 ± 0.014), suggesting that toothless geometries are inherently more stable for AI-based segmentation. Conversely, fully dentate cases exhibited slightly lower accuracy (DSC 0.936 ± 0.030 for Class V modifications), indicating that removing teeth and modifying the alveolar ridge in highly dentate cases introduces more anatomical variability. However, even in these cases, segmentation remained within clinically acceptable accuracy ranges. Thus, both versions could be used with sufficient accuracy. The Cl. III version is better suited for surgical planning with scapular or iliac crest flaps, as opposed to the thinner class V representing a better correspondence with the shape of a fibular transplant with its thinner tube shape structure. The geometries are sufficiently close to the original in regions that are supposed not to be modified, i.e. the basal part of the mandibular body and the ascending branches (H2). The caudolateral curve analysis confirmed that baseline mandibular morphology was preserved, supporting the anatomical validity of AI-generated modifications. This is particularly important in reconstructive surgery, where deviations in basal mandibular structure could compromise the accuracy of bony transplants and dental implant positioning.

The original data generally yields higher accuracy than the modified datasets, particularly when considering the performance of Class V models, confirming our hypothesis (H3). In this study, a more carefully curated subset of the data from [9] was used (e.g. omitting incoherent surface data or faulty segmentations). In combination with using a different implementation of the underlying UNet architecture, the results from this study showed overall higher accuracies than the ones previously reported for the segmentation of mandibles with teeth in [9] (Dice Coefficient 0.9482).

Verhelst et al. report an average Dice score of 0.9722 for the segmentation of mandibles without tooth crowns (similar to our Class III) from CBCT imaging [11]. Ilesan et al. investigated commercially available mandible segmentation models that reached average Dice scores between 0.912 and 0.949 for the mandible bone and 0.930 to 0.938 for the full mandible including teeth on a dataset consisting of ten CT and ten CBCT scans [20]. The DentalSegmentator model [21] showed a Dice score of 0.945 on a mixed CT and CBCT dataset and a score of 0.962 on another CBCT-only dataset for segmentation of the mandible bone.

Limitations of the work are the distinct focus on a very specific application with the aim of generating representation of edentulous mandibles for the use in surgical planning of mandibular reconstruction with specific transplants. Thus, the potential for generalization to other anatomical regions and use in other medical domains remains speculative, yet promising.

Potentially, results may further be improved, especially in the case of small sample sizes with the application of expansion transfer learning (ETL), that indicate to be advantageous in certain settings in relation to multi label segmentation of the carpal bones from magnetic resonance imaging data [22].

While the present study demonstrates promising accuracy and anatomical validity in generating edentulous mandibular geometries from dentate CT data, future work should incorporate clinical validation. In addition, future work should focus on expanding the dataset to include multi-center sources to improve generalizability and reduce institutional bias. Incorporating annotations from multiple independent raters would also enable assessment and enhancement of inter-rater reliability in ground-truth segmentation.

## Conclusion

This study demonstrates that AI-driven segmentation can generate anatomically valid edentulous mandibles from dentate CT scans with high accuracy. The proposed approach successfully removes dental geometries while preserving key mandibular structures, making it suitable for reconstructive surgical planning. Thus, our findings confirm that AI models can estimate geometrically accurate toothless mandibles while maintaining individual anatomical characteristics.

This method enhances virtual surgical planning by automating the adaptation of alveolar ridge geometry, reducing manual effort, and improving precision. While modified datasets showed slightly lower accuracy than original ones, overall performance remained within clinically acceptable limits. The study also highlights the advantages of single-label models in segmentation accuracy, though multi-label models offer higher efficiency.

By integrating AI into digital surgical workflows, this approach has the potential to improve planning accuracy and patient outcomes in mandibular reconstruction. Future research should explore its applicability to other anatomical structures and refine the models using larger datasets.

## Materials and methods

In this work, a two-stage segmentation approach is proposed to segment the bony mandible, as described in the related publication [9, 23]. It is adapted to the special needs for the modified task of generating toothless mandibles based on individual CT data.

### Available imaging data

The original data that was the basis of the present work was used in previous studies of our group [24–26]. Exclusion criteria were bony defects of the mandible, apparent malformation, low quality of the imaging data, and insufficient quality of the manually generated ground truth segmentations. Thus, from an initial collective of 509 a subset of 246 sets of data was included in this study. Dental status was protocoled from visual inspection of the virtual data after application of exclusion criteria and showed cohort sizes of 55 fully dentate mandibles, 136 partially dentate mandibles and 55 completely edentulous mandibles.

We confirm that all methods were carried out according to the applicable guidelines and regulations and institutional approval (EK 260/20) of the Independent Ethics Committee of the Faculty of Medicine of RWTH Aachen University Hospital was obtained. Due to the retrospective nature of the study, the Independent Ethics Committee of the Faculty of Medicine of RWTH Aachen University Hospital waived the need to obtain informed consent.

### Data preparation

Edentulous mandibles are typically classified according to their dental status and the degree of atrophy with the scheme according to Cawood and Howell [19] into six categories.

**Fig. 6:**
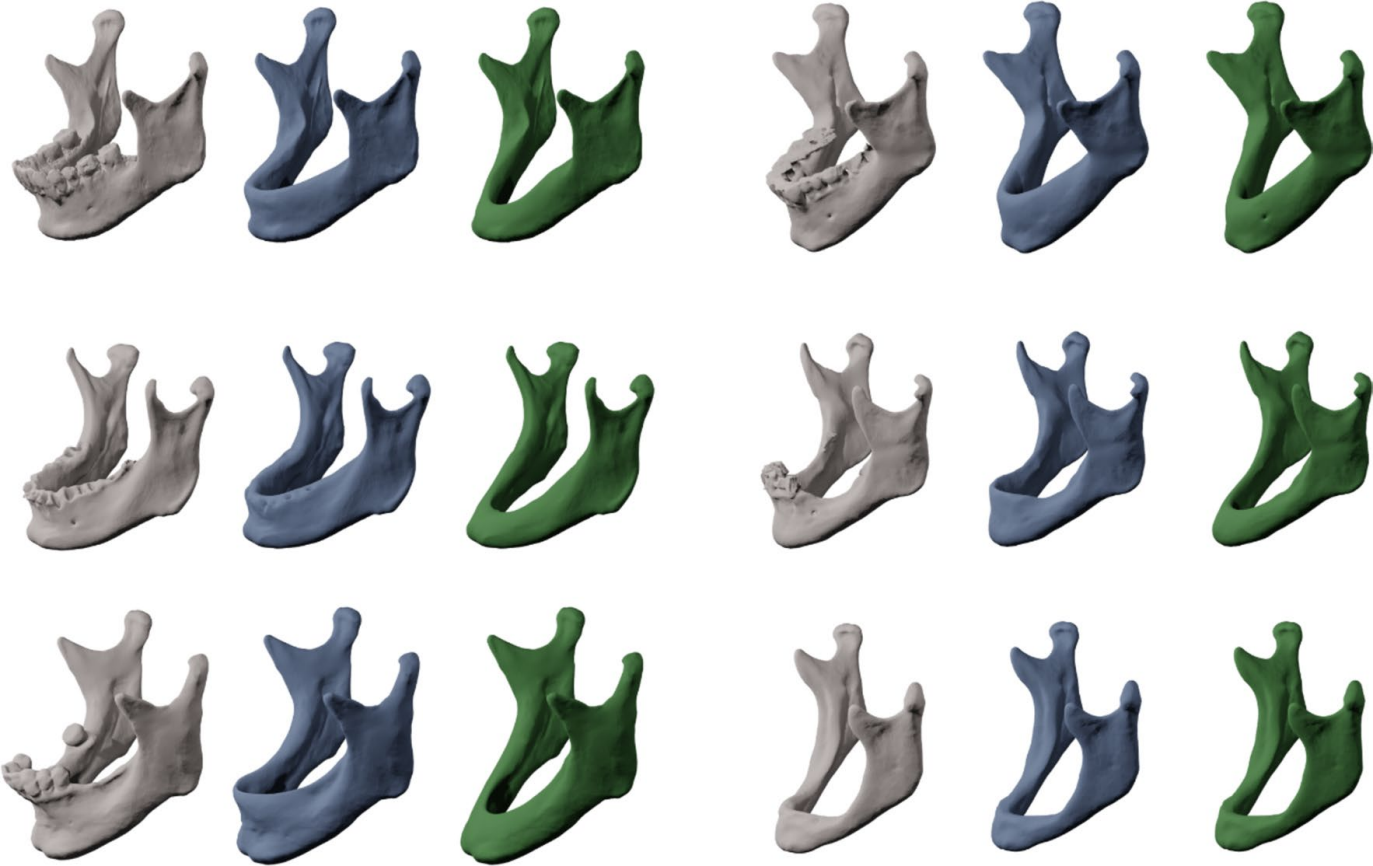
Six sets of corresponding examples of ground truth segmentations. Gray: original datasets (O), blue: toothless datasets (Cl. III) and green: (Cl. V). The general shape of these mandibles remains unchanged, but the dentate part is manually removed (Cl. III) and the alveolar ridge further reduced (Cl. V), respectively

The ground truth segmentations were based on a manual segmentation, threshold-based approach using Mimics (Materialise, Leuven, Belgium) [27, 28] and the data was stored as triangulated surfaces in STL file format. That data was manually altered to represent toothless data by removing digitally the tooth-bearing compartment with the modification tools of the 3D software Blender (version 2.83.0, Blender Foundation, Amsterdam, The Netherlands). Two different versions were generated from these data according to the six-category classification of Cawood and Howell [19], (a) a moderate reduction of the alveolar crest, resembling a class III [19] and (b) a more aggressive reduction of the alveolar crest, resembling a class V atrophic mandible. The validity of this classification could be confirmed in extensive virtual examinations of CT data of mandibles [24]. Thus, generating three different sets of data, abbreviated in the following sections as original (O), class III (Cl. III) and class V (Cl. V) (Fig. 6). The preparation of that data was manually performed by one experienced person and checked by an experienced professional for anatomical validity.

### Pipeline for mandible segmentation

**Fig. 7:**
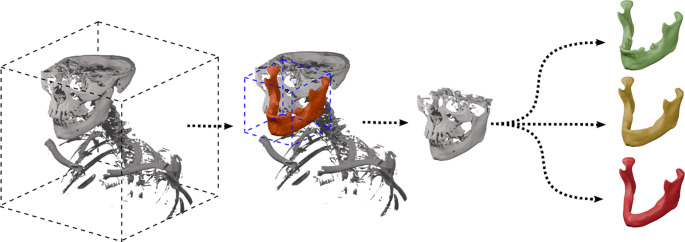
Two-stage pipeline for image segmentation: The whole field of view is used for the first stage (left) and the resulting segmentation (orange) is used to define a matching bounding box (blue) to derive a region of interest for a detailed segmentation with the different models deriving different dental status, i.e. like the original data (green), a class III edentulous (yellow) and a class V edentulous version (red), respectively

A two-stage approach is used, following the general layout described by previous publication by our group [9, 29]. In this approach, a first segmentation stage is used to detect the mandible in the complete field of view of the imaging data (Fig. 7). This initial stage is used only to find the region of interest for a more detailed segmentation in a second stage. To do so, we use the first stage segmentation result and use it for the generation of a bounding box to crop the whole imaging data around the detected region that is suspected to be the region of interest. This approach has shown its viability in a variety of applications in mandibular segmentation and beyond [9, 22, 29].

**Fig. 8:**
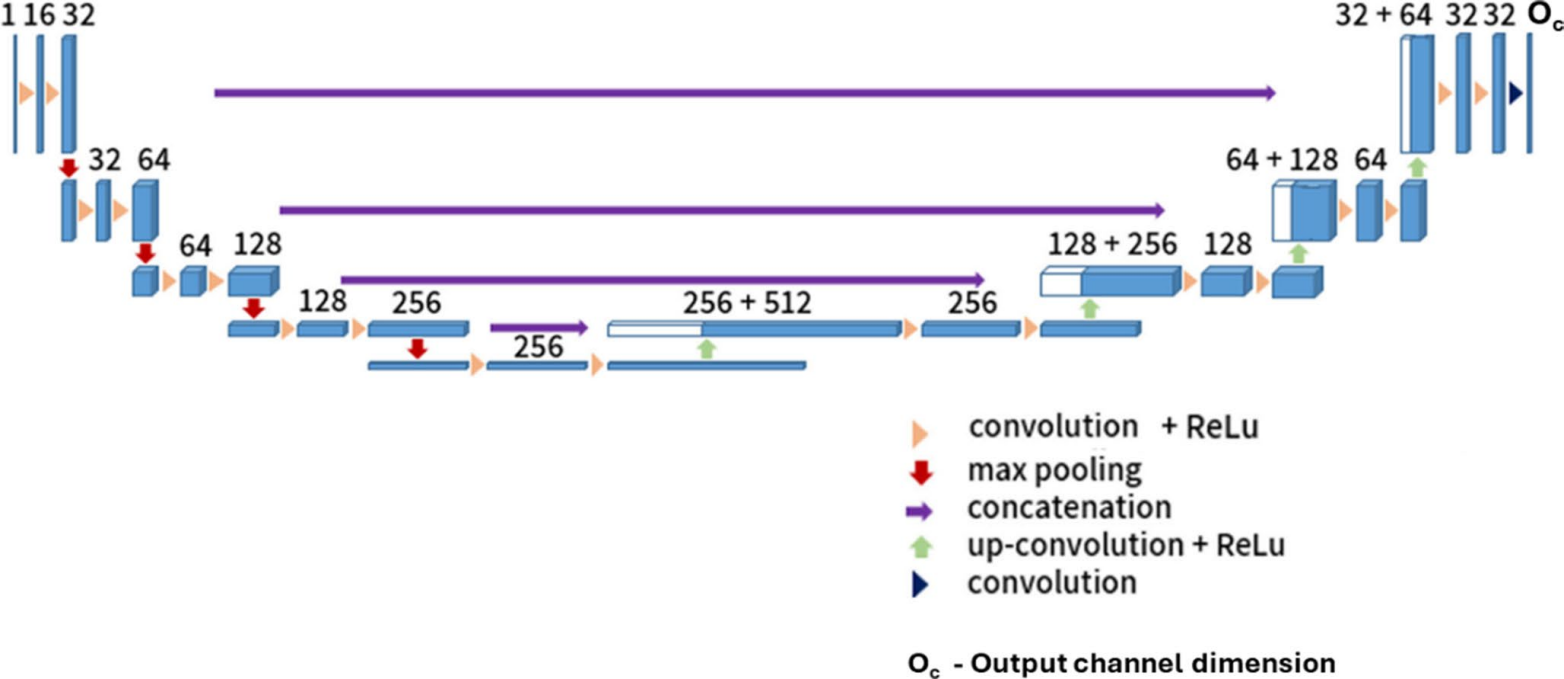
3D U-Net architecture (adapted from Pankert et al. [9]) used in the two-stage mandible segmentation pipeline. The same network architecture is used for both stages. In the first stage, and in each of the three single-label second-stage models (original, Class III, Class V), the network outputs a single binary channel (O_c_=1). In the multi-label second-stage model, the architecture remains unchanged except for the final layer, which outputs three independent binary channels (O_c_=3). All inputs are resampled to a resolution of 144 × 144 × 144 voxels with one intensity channel

For the training of the first stage, all volumetric data was resampled to a common resolution of 144 × 144 × 144 voxels. This segmentation is used as the basis for a more detailed segmentation of the mandible within a region of interest defined as the bounding box around the first stage result with a padding of 5 mm in all directions around the prediction of the first stage. Again, for this second stage, the cropped volumetric data was resampled to a resolution of 144 × 144 × 144, thus providing a higher relative resolution. If any predicted label voxels are detected within 5 voxels of the 144 × 144 × 144 volume’s border after the second stage, an iterative correction extracts a mesh from the voxel prediction and pads its bounds by 5 mm in world coordinates. This adjusted region is used as refined input for the second stage model again, iterating until the mesh bounds in world coordinates change by less than 2 mm or 20 iterations are reached. Figure 8. shows a schematic diagram of the 3D U-Net based segmentation network [9] used for both the first and second step of our segmentation pipeline.

All prepared pipelines use a first stage model trained on the original dataset, excluding the modified ground truths. This way, the bounding box (Fig. 7, blue), as defined by unmodified parts, such as chin, gonions, condyles and coronoid processes as well as the resampling are identical for all three different subsequent second stage models (O, Cl. III, Cl. V).

Data was split into train, validation and test data sets, with a ratio of 70%, 10% and 20%, respectively. This split was applied separately for all dental status categories (fully dentate, party dentate, and edentulous) to ensure all statuses are represented in each set. For all groups of datasets (O, Cl. III, Cl. V) the split was identical, thus having corresponding train, validation and test sets, to avoid any bias (Table 4).

**Table 4:** Number of cases by category and training split

Category	Total	Train	Validation	Test
Full	55	39	5	11
Partly	136	96	14	26
Edentulous	55	39	5	11
Total	246	174	24	48

Hence, the training pipeline yielded five different models that were trained in completely separate runs:

- One Model for the first step (trained on the O dataset in full resolution).
- Three different single label models for the second step (trained on the respective dataset cropped around the padded ground truths) for the three groups O, Cl. III and Cl. V.
- One combined model with three partially overlapping output channels, thus O, Cl. III and Cl. V were regarded as different output channels of the same model.

### Augmentation

For the improvement of robustness and performance of the training, an augmentation pipeline based on transformations from MONAI [30] was used that was evaluated within a structured ablation study during preliminary research after establishing our initial mandibular segmentation model [9].

For the training process, we applied the following combined augmentations: The first-step model employed elastic deformation with a magnitude range of 250–1000 and the second-step models with a magnitude range of 1000–2500, both respectively with a sigma range of 10–13. All models incorporated random rotations (−20° to 20°), translations (−30 to 30 voxels), scaling (0.75 to 1.25), and mirroring along the left-right axis. All augmentations were applied independently with a 50% probability. Gaussian Noise and Gaussian Blur were excluded from the final pipeline as they showed no performance improvements in our initial ablation studies.

### Post-Processing

After the actual pipeline of segmentation, the predictions in the standard resolutions (144 × 144 × 144) were resampled to original resolution of each imaging dataset, respectively, for subsequent evaluations. Then, this binary volumetric data at the original imaging resolution was further post-processed to allow for a representation of the acquired data as a triangulated surface mesh, by using the marching cubes algorithm [31].

Smoothing was performed using Taubin filtering [32] with default parameters (λ = 0.5, ν = 0.5) and 100 iterations from the geometry library trimesh [33]. Taubin filtering overcomes the shrinkage issue of standard Laplacian smoothing and effectively reduces noise while preserving essential geometric features.

### Used software and hardware

The proposed approach was implemented in Python (version 3.10) and PyTorch (version 2.1.2).

Trainings and evaluations conducted on a computer system with the following specifications: Processor: Intel(R) Xeon(R) Gold 5122 CPU @ 3.60 GHz RAM: 128.0 GB Graphics Card: NVIDIA Quadro RTX 5000 memory size 16GB. Operating System: Ubuntu 22.04.3 LTS.

### Study design

For the validation of the segmentation performance, the processed AI-generated segmentations were quantitatively compared to their corresponding ground truth surfaces using established evaluation metrics. The accuracy of segmentation was assessed through direct comparisons between predictions (AI segmentations) and ground truths, considering three groups:

- O: Original dataset segmentation.
- Cl. III: Class III alveolar ridge segmentation.
- Cl. V: Class V atrophic mandible segmentation.

These comparisons were conducted in two configurations:

(A) Across the entire test dataset (*n* = 48).
(B) Subdivided according to dental status (fully dentate: *n* = 11, partially dentate: *n* = 26, edentulous: *n* = 11).

**Fig. 9:**
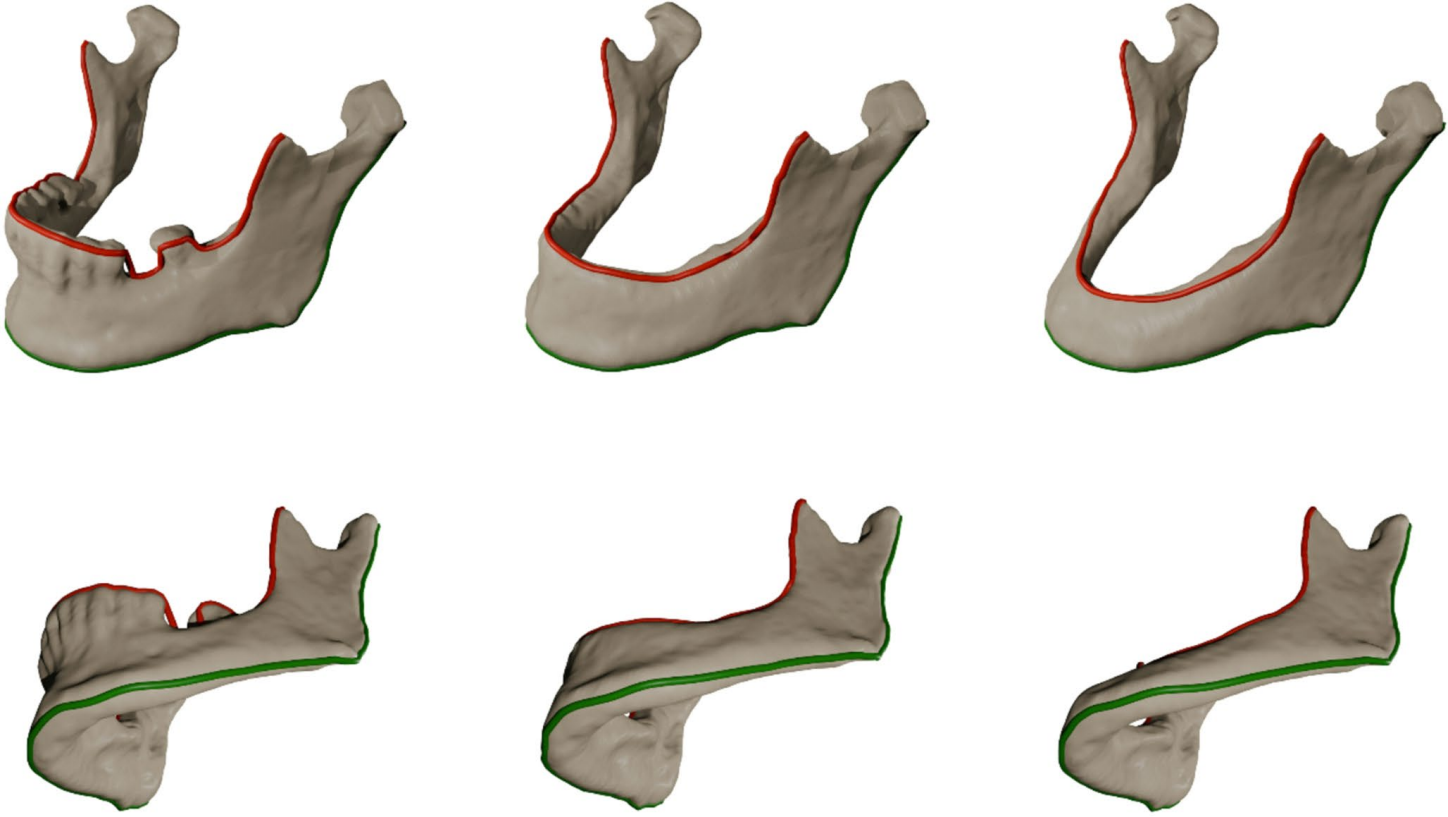
Visualization of automatically detected anatomical curves: dental curve (red) and caudolateral curve (green) for original data, modified Class III and modified Class V

Additionally, curvature metrics were introduced to quantify the shape of the bone curvature along specific anatomical regions. First, the caudolateral curve [34] was analyzed across the different segmentation variations (O, Cl. III, Cl. V). Ideally, this curve should remain consistent across all variations, as the basal shape of the mandible is not expected to change due to the removal of dentition [35]. Secondly, a curvature metric following the tops of the tooth cusps or the alveolar crest in edentulous mandibles was automatically generated in a similar approach in order to detect its anatomical shape in an objective and reproducible way (referred to as dental curve). This metric is expected to change significantly between different groups (O, Cl. III, Cl. V) but may be used to evaluate the ability of the models to detect the desired degree of alveolar resorption (Fig. 9). Both of these evaluations served as an additional anatomical validity check to ensure that smoothing did not introduce distortions in regions that should remain unchanged.

The final assessment of segmentation accuracy was done in a volumetric comparison with Dice similarity coefficient (DSC) and based on surface deviation metrics, by symmetric average 3D surface distance (ASD).

The DSC between two meshes A and B is defined as:

$$\:DSC\left(A,B\right)=\frac{2|A\cup\:B|}{\left|A\right|+\left|B\right|}$$

Where $\:A\cup\:B\:$ is the Boolean intersection between the meshes and $\:\left|M\right|$ is the volume of a mesh.

The symmetric ASD is defined as:

$$\:ASD\left(A,B\right)=\frac{1}{2}(dASD\left(A,B\right)+dASD(B,A\left)\right)$$

where the directed average surface distance is:

$$\:dASD\left(A,B\right)=\frac{1}{\left|{V}_{A}\right|}\sum\:_{v\in\:{V}_{A}}dist(v,B)$$

Here, $\:{V}_{A}$ is the set of vertices of $\:A$ and $\:dist(v,B)$ is the unsigned minimum distance between a vertex $\:v$ and the surface of mesh $\:B$.

Both metrics were computed using trimesh [36].

These metrics provided a comprehensive evaluation of the smoothing process and its impact on anatomical fidelity in the context of AI-assisted mandibular reconstruction.

In all metric evaluations a comparison between the prediction results of the full pipeline (both stages) on the test dataset was performed against the corresponding ground truth data.

Additionally, for visual assessment of the performance of the models in different regions of the mandible, color-coded visualizations of the mandibular data were performed against the original datasets.

### Statistical evaluation

For statistical evaluation of differences among the groups, after checking for normal distribution with Shapiro–Wilk test, non-parametric statistical tests were employed to assess differences between groups. Specifically, the Wilcoxon signed-rank test was used for paired comparisons, and the Mann-Whitney U test was applied for independent group comparisons. A significance threshold of *p* = 0.05 was defined to determine statistical significance. To account for multiple comparisons, the Bonferroni-Holm correction was applied. Statistical computations were conducted to validate the observed differences in Dice similarity coefficients and average surface distances. All statistical analyses were performed using the SciPy library in Python.

## Data Availability

No datasets were generated or analysed during the current study.
